# Delivery of Therapeutic RNA to the Bone Marrow in Multiple Myeloma Using CD38‐Targeted Lipid Nanoparticles

**DOI:** 10.1002/advs.202301377

**Published:** 2023-05-12

**Authors:** Dana Tarab‐Ravski, Inbal Hazan‐Halevy, Meir Goldsmith, Lior Stotsky‐Oterin, Dor Breier, Gonna Somu Naidu, Anjaiah Aitha, Yael Diesendruck, Brandon D. Ng, Hagit Barsheshet, Tamar Berger, Iuliana Vaxman, Pia Raanani, Dan Peer

**Affiliations:** ^1^ Laboratory of Precision NanoMedicine The Shmunis School of Biomedicine and Cancer Research George S. Wise Faculty of Life Sciences Tel Aviv University Tel Aviv 69978 Israel; ^2^ Department of Materials Sciences and Engineering Iby and Aladar Fleischman Faculty of Engineering Tel Aviv University Tel Aviv 69978 Israel; ^3^ Center for Nanoscience and Nanotechnology Tel Aviv University Tel Aviv 69978 Israel; ^4^ Cancer Biology Research Center Tel Aviv University Tel Aviv 69978 Israel; ^5^ Department of Pharmacology Weil Cornell Graduate School for Biomedical Sciences New York NY 10065 USA; ^6^ Department of Immunology Sloan Kettering Institute Memorial Sloan Kettering Cancer Center New York NY 10065 USA; ^7^ Institute of Hematology Rabin Medical Center Beilinson Hospital Petah Tikva 4941492 Israel

**Keywords:** BM niche, cancer, lipid nanoparticles, MM murine model, multiple myeloma, RNA therapy, targeted delivery

## Abstract

Multiple myeloma (MM) is a cancer of differentiated plasma cells that occurs in the bone marrow (BM). Despite the recent advancements in drug development, most patients with MM eventually relapse and the disease remains incurable. RNA therapy delivered via lipid nanoparticles (LNPs) has the potential to be a promising cancer treatment, however, its clinical implementation is limited due to inefficient delivery to non‐hepatic tissues. Here, targeted (t)LNPs designed for delivery of RNA payload to MM cells are presented. The tLNPs consist of a novel ionizable lipid and are coated with an anti‐CD38 antibody (*α*CD38‐tLNPs). To explore their therapeutic potential, it is demonstrated that LNPs encapsulating small interference RNA (siRNA) against cytoskeleton‐associated protein 5 (CKAP5) lead to a ≈90% decrease in cell viability of MM cells in vitro. Next, a new xenograft MM mouse model is employed, which clinically resembles the human disease and demonstrates efficient homing of MM cells to the BM. Specific delivery of *α*CD38‐tLNPs to BM‐residing and disseminated MM cells and the improvement in therapeutic outcome of MM‐bearing mice treated with *α*CD38‐tLNPs‐siRNA‐CKAP5 are shown. These results underscore the potential of RNA therapeutics for treatment of MM and the importance of developing effective targeted delivery systems and reliable preclinical models.

## Graphical abstract

Targeted LNPs composed of a novel lipid to deliver therapeutic RNA for multiple myeloma (MM) treatment are generated. First, siRNA silencing the expression of CKAP5 is encapsulated and decreases cell viability of MM cells. Next, LNPs distribution to MM cells in the bone marrow is validated and their efficacious therapeutic outcomes are shown in a newly established MM mouse model.

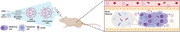

## 1 Introduction

Multiple myeloma (MM) is a plasma‐cell malignancy in which malignant differentiated plasma cells proliferate and metastasize primarily in the bone marrow (BM), and as the disease progresses, in the peripheral blood and other extramedullary sites.^[^ ^1^ ^]^ It is the second most common hematological malignancy and is usually diagnosed in people over 60 years old. The clinical symptoms of MM include hypercalcemia, renal insufficiency, anemia, and lytic bone lesions, collectively known as CRAB features. These clinical manifestations are a result of the extensive secretion of the monoclonal immunoglobulin protein (M‐protein), colonization of the MM cells in the bone marrow, and the elaborate interactions between MM cells with the bone marrow microenvironment.^[^ ^2^ ^]^ The survival rate of MM disease has significantly improved over the last few years due to the development of novel anti‐cancer drugs, however, MM is still considered incurable as patients eventually relapse and develop drug resistance. For this reason, there is a constant need for expanding the available therapeutic arsenal for this disease.

The development of effective treatments for MM greatly relies on reliable murine models that both resemble the human MM disease and allow the evaluation of treatment capability to reach and affect MM cells in the BM niche.^[^ ^3^, ^4^ ^]^ Today, most preclinical xenograft murine models are established by a subcutaneous or intravenous injection of human MM cell lines into mice. As a result, the cells home to non‐related organs such as the skin, liver, and lungs, but lack both BM colonization and interactions with the BM microenvironment. Generation of new models that overcome these limitations of poor engraftment to the BM and clinical similarity to the human disease is therefore crucial for drug development for MM.

RNA‐based therapeutics are powerful and clinically approved therapeutic tools, potentially capable of inducing gene silencing, editing, or expression in any cell.^[^ ^5^ ^]^ However, applying RNA therapy for B‐cell malignancies is extremely challenging as lymphocytes are generally resistant to in vivo transfection with RNA molecules.^[^ ^6^, ^7^, ^8^ ^]^ Lipid nanoparticles (LNPs) have evolved dramatically over the last few years and are today the most advanced non‐viral delivery strategy for RNA due to their minimal toxicity, low batch‐to‐batch variation, and efficient encapsulation of RNA.^[^ ^9^, ^10^, ^11^, ^12^ ^]^ Ionizable cationic lipids constitute an essential part of LNPs and greatly determine their organ distribution and transfection capabilities to different cell types.^[^ ^13^, ^14^ ^]^ While attempts to systemically deliver RNA encapsulated within LNPs have succeeded to the liver, reaching extrahepatic tissues, such as the BM, further complicates their translation for nonhepatic applications.^[^ ^15^ ^]^ Furthermore, harnessing LNPs for delivery of therapeutic RNA to MM cells may also necessitate employing a targeting moiety to generate targeted LNPs (tLNPs) and facilitate their specific delivery and internalization.^[^ ^8^, ^16^ ^]^ Hence, comprehensive research is necessary for developing an efficient and safe delivery strategy that will allow the application of RNA therapy for MM disease and other B‐cell malignancies.

Herein, we report on the generation of functional tLNPs which encapsulate siRNA and are coated with an anti‐CD38 antibody (*α*CD38‐tLNPs). We evaluate their biodistribution and therapeutic effects on MM cells both in vitro and in vivo and use a new xenograft MM mouse model to confirm their arrival to the BM niche and clinical relevance.

## 2 Results

### 2.1 Screening and Characterization of LNPs for Efficient Delivery to Human MM Cells

**Figure 1:**
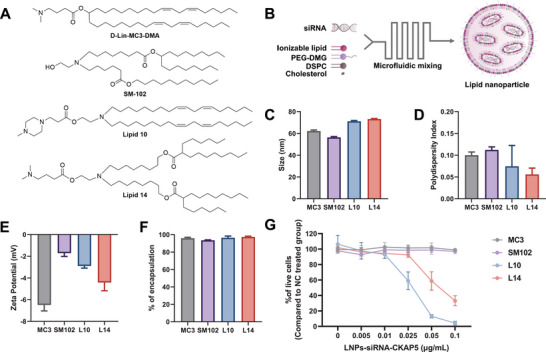
Screen of ionizable cationic lipids for the transfection of human MM cells. A) Chemical structures of D‐Lin‐MC3‐MC3‐DMA, SM102, and the selected ionizable cationic lipids from the lipid library. B) Schematic illustration of LNPs preparation. C) LNPs mean diameter (nm); D) polydispersity index (PDI). E) *ζ* potential (mV), as measured by Zeta Sizer. F) Percentage of encapsulation efficiency as measured by a RiboGreen assay. G) Percentage of CAG cell viability 72 h post‐transfection with either PBS or different concentrations of siRNA‐CKAP5 (0.005–0.1 µg mL^−1^ of total RNA) as measured by XTT assay. Cell viability percentages were normalized to cells treated with 0.1 µg mL^−1^ of total RNA of LNPs‐siRNA‐NC. Data are means of ±SD of three independent experiments.

To achieve an efficient delivery to MM cells within the bone marrow, we previously screened a library of proprietary ionizable amino lipids to determine which is the most efficient for transfection of MM cells.^[^ ^17^ ^]^ Out of the screen, lipid 10 and lipid 14 were chosen as the two lead candidates and were further compared to DLin‐MC3‐DMA and SM‐102, two ionizable cationic lipids which are FDA‐approved for delivery of RNA^[^ ^10^, ^12^ ^]^ (**Figure** **1**A). LNPs were prepared according to the previously described method^[^ ^17^ ^]^ (Figure 1B) and found to be uniform in size with a diameter of 56–73 nm, polydispersity index of 0.05–0.11, and *ζ* potential ranging between (‐1.7)‐(‐6.4) mV as measured by dynamic light scattering (Figure 1C–E). The encapsulation efficiency of the siRNA was similarly high in all LNPs (>95%) (Figure 1F). As proof of concept for the generation of LNPs that can successfully transfect human MM cells, we evaluated the in vitro therapeutic effect of the LNPs on the human MM CAG cell line by encapsulating either an siRNA‐NC as negative control or an siRNA that silences the expression of cytoskeleton‐associated protein 5 (CKAP5) and determining cell death.^[^ ^18^ ^]^ CKAP5 is a cytoskeleton‐binding protein that binds to the plus ends of microtubules and regulates bipolar spindle formation and centrosomal organization during mitosis.^[^ ^19^, ^20^ ^]^ It is overexpressed in many types of cancers and was identified as a potentially druggable target for MM.^[^ ^18^, ^20^ ^]^ Only LNPs composed of lipid 10 and lipid 14 induced cell death in a dose‐dependent manner, with lipid 10 being the most efficient as cell viability dropped to 4.17% after incubation for 72 h with L10‐LNPs‐siRNA containing a total RNA concentration of 0.1 µg mL^−1^ (Figure 1G). However, at the same conditions, LNPs composed of DLin‐MC3‐DMA or SM‐102 did not induce cell death in MM cells. The activity of L10‐, L14‐, MC3‐ and SM102‐based LNPs encapsulated with siRNA‐CKAP5 was also tested on the human ovarian cancer Ovcar8 cell line and the human colon cancer HCT116 cell line. No difference was found between the LNP formulations and all of them induced cell death in both the Ovcar8 and the HCT116 cell lines (Figure S1, Supporting Information), indicating the advanced ability of L10‐LNPs‐siRNA for transfecting human MM cells in vitro. Based on these results, we chose L10‐LNPs‐siRNA as the lead candidate for the continuation of this study.

### 2.2 LNPs‐siRNA‐CKAP5 Induce an Efficient Silencing of CKAP5, Leading to Cell Cycle Arrest in MM Cells In Vitro

**Figure 2:**
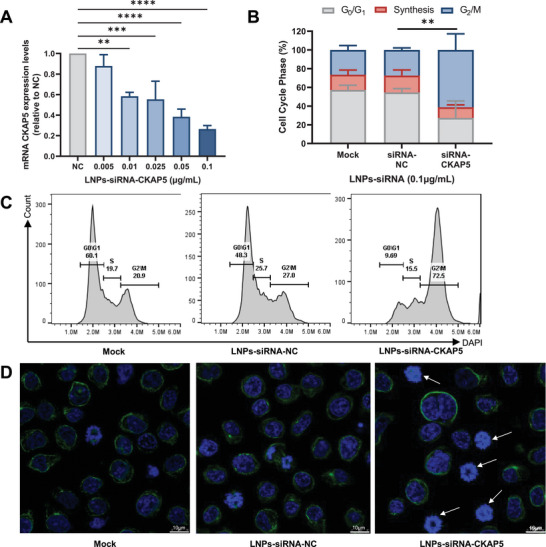
Therapeutic effects of LNPs‐siRNA‐CKAP5 on CAG cells in vitro. A) mRNA expression levels of CKAP5 in CAG cells 24 h post‐transfection with different concentration of LNPs‐siRNA‐CKAP5 (0.005‐0.1 µg mL^−1^ of total RNA) or 0.1 µg mL^−1^ total RNA of LNPs‐siRNA‐NC. LNPs‐siRNA‐NC‐treated cells were used to determine the basal CKAP5 expression levels. B) Cell cycle analysis by flow cytometry of CAG cells 36 h after treatment with mock, LNPs‐siRNA‐NC, and LNPs‐siRNA‐CKAP5 in concentration of 0.1 µg mL^−1^ total RNA. Bar charts represent the percentage of G_0_/G_1_, synthesis, and G_2_/M cell cycle phases. Representative cell cycle arrest C) diagram and D) confocal microscopy of CAG cells 36 h post transfection with mock, LNPs‐siRNA‐NC, and LNPs‐siRNA‐CKAP5 in concentration of 0.1 µg mL^−1^ total RNA. For confocal microscopy, DNA was stained with Hoechst 33342 (blue) and *α*Tubulin was stained with a secondary antibody conjugated to Alexa488 (green). Data in A,B are means of ±SD of three independent experiments. One‐way analysis of variance (ANOVA) with Tukey multiple comparison test was used to assess the significance. **P < 0.01, ***P < 0.001, ****P < 0.0001.

To validate the mechanisms by which cell death is caused in MM cells as witnessed previously (Figure 1G), human MM cell lines were transfected and evaluated for silencing and effect on cell cycle. Twenty‐four hours after transfecting human MM CAG cell line with LNPs‐siRNA‐CKAP5, a dose‐dependent reduction in the mRNA levels was observed by qRT‐PCR and achieved maximal levels of 73.5% reduction with LNPs containing total RNA of 0.1 µg mL^−1^ (**Figure** **2**A). A significant G2‐M arrest with an average of 62.4% cells stuck during this phase was witnessed by flow cytometry 36 h post‐transfection, while control LNPs‐siRNA‐CKAP5 had no effect on cell cycle profile (Figure 2B,C). The disruption caused by the knockdown of CKAP5 was also visualized by confocal microscopy, and significantly more cells treated with LNPs‐siRNA‐CKAP5 appeared to be arrested during mitosis (Figure 2D). These findings, combined with the effect of on cell viability, confirm the mechanism of action and therapeutic effect of LNPs‐siRNA‐CKAP5 on MM cells in vitro.

### 2.3 Generation of Anti‐CD38 Targeted LNPs

Following the screening of different ionizable cationic lipids and choosing to focus on L10‐LNPs‐siRNA, we generated targeted LNPs. Incorporation of a targeting moiety to LNPs can significantly enhance the efficiency and specificity of the delivery to hard‐to‐transfect cells, such as lymphocytes.^[^ ^8^ ^]^ We chose to target CD38, a glycoprotein which is overly expressed upon MM cells and many other B‐cell lymphoma cells such as mantle cell lymphoma.^[^ ^21^, ^22^ ^]^ CD38 was also shown to be clinically relevant for MM with Daratumumab, the first monoclonal antibody approved for treatment of MM.^[^ ^23^ ^]^

**Figure 3:**
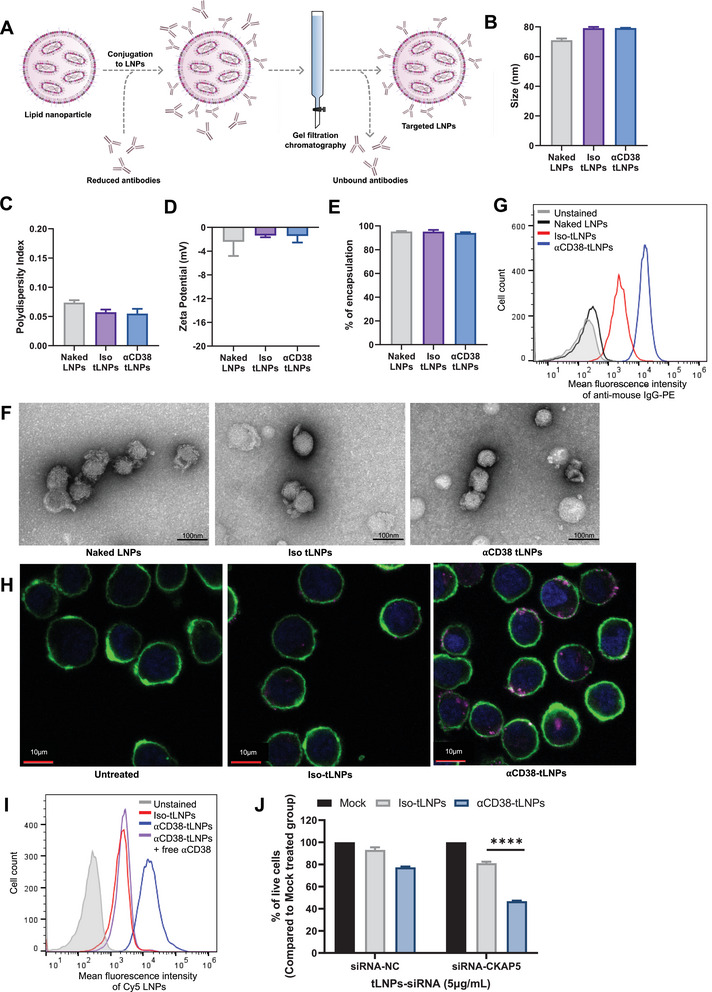
Characterization and evaluation of anti‐CD38 targeted LNPs. A) Schematic illustration of tLNPs preparation. B) Naked and targeted LNPs mean diameter (nm); C) polydispersity index (PDI). D) *ζ* potential (mV), as measured by Zeta Sizer. E) Percentage of encapsulation efficiency as measured by a RiboGreen assay. F) Representative transmission electron microscopy of naked and tLNPs. Experiment was repeated three times independently (Scale bar = 100 µm). G) In vitro binding of naked, iso‐tLNPs, and *α*CD38‐tLNPs to CAG cells. H) Representative live confocal images showing the internalization of *α*CD38‐tLNPs‐Cy5‐siRNA (shown in magenta) into CAG cells. DNA was stained with Hoechst 33342 (blue) and membranes were stained with an anti‐CD44 antibody conjugated to Alexa488 (green). (Scale bar = 10 µm). I) Competitive in vitro binding of iso‐tLNPs and *α*CD38‐tLNPs to CAG cells and binding of *α*CD38‐tLNPs to CAG cells pre‐coated with a free anti‐CD38 antibody. J) Representative XTT cell viability assay of primary MM cells treated with mock, iso‐tLNPs, or *α*CD38‐tLNPs encapsulating either siRNA‐NC or siRNA‐CKAP5 in a concentration of 5 µg mL^−1^ total RNA. Bar chart representing % of cell viability normalized to mock‐treated cells. Two‐way analysis of variance (ANOVA) with Tukey multiple comparison test was used to assess the significance. ****P < 0.0001.

To generate targeted LNPs (tLNPs), L10‐LNPs were conjugated to an anti‐CD38 (*α*CD38) antibody using maleimide‐thiol chemistry (**Figure** **3**A). After tLNPs preparation, conjugation, and purification the size, uniformity, *ζ* potential, and encapsulation efficiency of the LNPs were evaluated and no significant changes were observed (Figure 3B–E). The uniformity of the tLNPs before and after conjugation was also confirmed by transmission electron microscopy (Figure 3F).

Binding and internalization of *α*CD38‐tLNPs to the MM cells in vitro was witnessed by flow cytometry and confocal microscopy, respectively, using encapsulated fluorescently labeled Cy5‐siRNA (Figure 3G,H). As a control for the non‐specific binding of chemically conjugated tLNPs, L10‐tLNPs were conjugated to an isotype control IgG antibody (iso‐tLNPs). For evaluation of internalization, CAG cells were incubated for 30 min with either iso‐tLNPs or *α*CD38‐tLNPs and washed to allow internalization of bound LNPs only. The specificity to bind human MM cells in vitro via the CD38 receptor was confirmed by pre‐coating the cells with free unlabeled *α*CD38 antibodies prior to the addition of *α*CD38‐tLNPs and observing a decrease in the binding intensity of the LNPs (Figure 3I). Internalization of *α*CD38‐tLNPs to MM cells was also verified with primary MM cells extracted from the BM of MM patients. Internalized Cy5‐siRNA was detected only with the *α*CD38‐tLNPs, but not with iso‐tLNPs (Figure S2A, Supporting Information). To confirm the effects of the *α*CD38‐tLNPs on the therapeutic outcome, *α*CD38‐ or iso‐tLNPs encapsulating siRNA‐CKAP5 composed of lipid 10 were used to transfect primary MM cells ex vivo. The therapeutic effect of naked LNPs was not evaluated on primary MM cells since naked LNPs are unable to internalize into and transfect primary MM cells (Figure S2B,C, Supporting Information). While *α*CD38‐tLNPs‐siRNA‐CKAP5 induced 53.19% cell death of primary MM cells 72 h post‐tLNPs transfection, no notable therapeutic effect was visible in cells treated with either iso‐tLNPs‐siRNA‐CKAP5 or *α*CD38‐tLNPs‐siRNA‐NC (Figure 3J). The same effect was witnessed upon transfection of primary MM cells with *α*CD38‐ or iso‐tLNPs encapsulating siRNA to silence induced myeloid leukemia cell differentiation protein 1 (MCL1), where only *α*CD38‐tLNPs‐siRNA‐MCL1‐treated cells demonstrated a significant decrease in cell viability (Figure S2D, Supporting Information).

### 2.4 Establishment of Novel Xenograft MM Mouse Model

**Figure 4:**
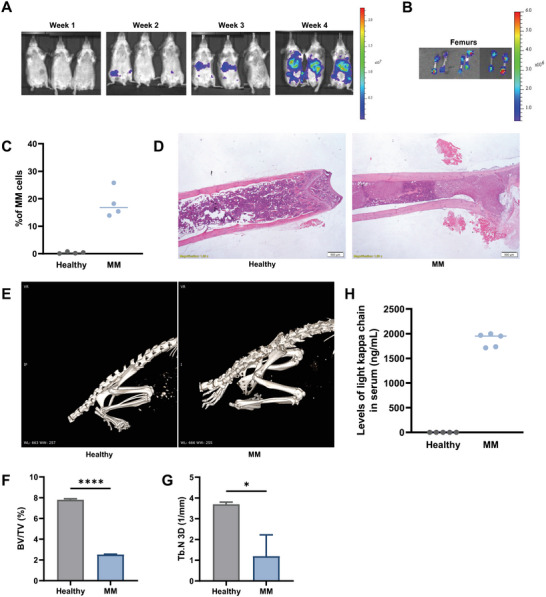
Establishment of novel xenograft MM mouse model. A) Tumor growth progression in mice injected via the caudal artery with CAG cells constitutively expressing luciferase (CAG‐Luc), monitored by IVIS in vivo bioluminescence imaging system. B) Bioluminescence imaging of the femurs 24 d after injection with CAG‐Luc cells to mice. C) Percentages of MM cells engraftment in the femurs 24 d post CAG‐Luc injection. Representative images of healthy (left) and MM‐bearing mice of D) H&E staining, and E) whole‐body CT, 24 d post‐tumor inoculation. Micro‐CT analysis of F) trabecular cone volume (BV/TV) and G) trabeculae per unit length (Tb.N 3D 1 mm^−1^) from healthy and MM‐bearing mice 24 d post injection of CAG‐Luc cells. Bar charts represent means ±SD. Data in A‐G are a representation of three independent experiments, *n* = 3 per group. H) Levels of light kappa chain monoclonal protein in the serum of healthy and MM‐bearing mice 24 d post tumor inoculation (ng mL^−1^), *n* = 5 per group and repeated 3 times. Unpaired T‐test was used to assess the significance. *P < 0.05, ****P < 0.0001.

An effective evaluation of the arrival of *α*CD38‐tLNPs to the MM cells in vivo greatly depends on the availability and reliability of an animal model. While there are many published xenograft MM mouse models, most of them are generated by subcutaneous or intravenous injection of the human MM cells and are therefore characterized by a lack of homing of MM cells to the BM and low resemblance to the human disease.^[^ ^24^, ^25^ ^]^ We established a novel xenograft MM mouse model by injecting CAG cells (1 × 10^6^) stably expressing luciferase via the tail caudal artery into 6‐ to 8 week old female R2G2‐SCID mice.^[^ ^26^ ^]^ The mice were weekly monitored by IVIS bioluminescent live imaging to follow disease progression (**Figure** **4**A) and after 24 d the mice were sacrificed and the liver, lungs, spleen, kidneys, and BM were harvested to assess the distribution of the MM cells. CAG‐Luc cells distributed to the liver and spleen (Figure S3A, Supporting Information), as with most xenograft MM models, but were also highly abundant in the BM (Figure 4B), with flow cytometry analysis showing an average engraftment of 15.8% in the BM at day 24 (Figure 4C and Figure S3B, Supporting Information). Furthermore, the tumors were confirmed to be inside the BM by H&E‐stained femoral slices (Figure 4D). In addition, MM‐bearing mice also displayed significant osteolytic bone lesions (Figure 4E), a decrease in trabecular bone volume (Figure 4F,G), and high levels of light kappa chain monoclonal protein in the serum (Figure 4H), similar to the human disease, thus highlighting the correlation between this newly established model and the clinical aspects of MM. An increased presence of osteoclasts, which are the major players in the BM microenvironment leading to bone destruction,^[^ ^1^, ^2^, ^27^ ^]^ was confirmed in the femurs of MM‐bearing mice by TRAP histology staining of the femurs (Figure S3C, Supporting Information).

### 2.5 Biodistribution of Anti‐CD38 Targeted LNPs to the BM In Vivo

**Figure 5:**
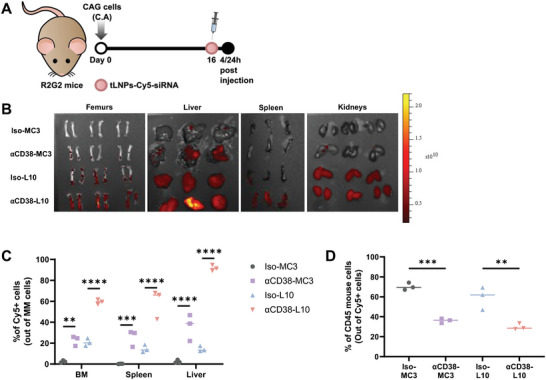
In vivo biodistribution of targeted LNPs in MM‐bearing mice. A) Schematic illustration of experimental design. B) Representative fluorescence imaging of femurs, liver, spleen, and kidneys 4 h after tLNPs injection, *n* = 3 per group. C) Percentages of Cy5‐positive MM cells in the BM, spleen, and liver 4 h after injection of tLNPs as analyzed by flow cytometry, *n* = 3 per group. D) Percentages of Cy5‐positive mouse CD45 cells in the BM 4 h after injection of tLNPs as analyzed by flow cytometry, *n* = 3 per group. One‐way and two‐way analysis of variance (ANOVA) with Tukey multiple comparison test was used to assess the significance. **P<0.01, ***P<0.001, ****P<0.0001.

Next, we evaluated the ability of *α*CD38‐tLNPs to deliver siRNA into MM cells residing inside the BM in our novel MM mouse model. We compared the biodistribution of tLNPs composed of L10, which we found to be effective for transfecting MM cells in vitro, with tLNPs composed DLin‐MC3‐DMA, as it is the only ionizable lipid approved for systemic delivery of RNA.^[^ ^10^ ^]^ Mice were injected with CAG‐Luc cells and 16 d post‐tumor inoculation were mock‐treated or treated retro‐orbitally with tLNPs loaded with fluorescently labeled siRNA coated with either an anti‐CD38 or isotype control antibody (**Figure** **5**A). The liver, spleen, kidneys, and BM were extracted 4 h later and analyzed by IVIS live imaging (Figure 5B) and flow cytometry for uptake of siRNA into MM cells and mouse CD45^+^ cells (gating strategy of human MM cells and mouse CD45^+^ cells appears in Figure S4A, Supporting Information). Fluorescently labeled siRNA was detected in 59.43% of MM cells in the BM in mice treated with *α*CD38‐L10‐tLNPs, compared to 22.67% with *α*CD38‐MC3‐tLNPs and 20.83% with iso‐L10‐tLNPs (Figure 5C). A similar outcome of favorable *α*CD38‐L10‐tLNPs accumulation in MM cells was also observed upon examination of the spleen and liver. Significant levels of siRNA were still found in MM cells in the BM 24 h after injection only in mice injected with *α*CD38‐L10‐tLNPs (Figure S4B, Supporting Information). Furthermore, significantly lower levels of siRNA were detected in mouse CD45^+^ cells in the BM of mice injected with *α*CD38‐tLNPs compared to iso‐tLNPs, further emphasizing the importance of employing anti‐CD38 antibody as a targeting moiety to decrease off‐target effect (Figure 5D). Lastly, an injection of tLNPs caused no elevation of the liver enzymes aspartate aminotransferase (AST), alanine transaminase (ALT), alkaline phosphatase (ALP), or gamma‐glutamyl transpeptidase (GGTP) (Figure S4C–F, Supporting Information).

### 2.6 Efficacy of Anti‐CD38 Targeted siRNA‐CKAP5 LNPs in MM‐Bearing Mice

**Figure 6:**
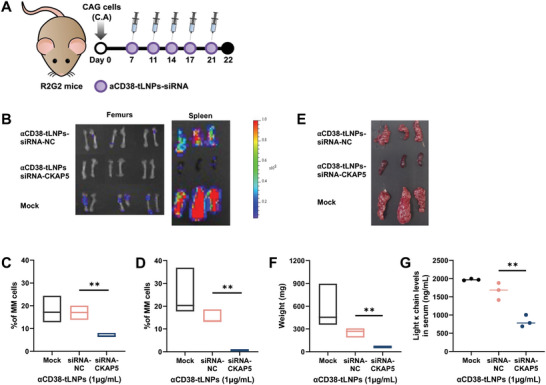
In vivo therapeutic effect of *α*CD38‐tLNPs‐siRNA‐CKAP5 in MM‐bearing mice. A) Schematic illustration of experimental design. B) Representative bioluminescence imaging of femurs and spleen 22 d after tumor inoculation. Percentages of MM engraftment in the C) BM and D) spleen as analyzed by flow cytometry. E) Representative images of the spleen at experiment termination. F) Weight of spleen 22 d post tumor inoculation (mg). G) Levels of light kappa chain monoclonal protein in the serum 22 d post tumor inoculation (ng mL^−1^). Each efficacy study was conducted with 3 mice per group. Data in A–G are a representation of three independent experiments. One‐way analysis of variance (ANOVA) with Tukey multiple comparison test was used to assess the significance. **P < 0.01.

To test whether the therapeutic potential of *α*CD38‐tLNPs can be translated to MM‐bearing mice in vivo, *α*CD38‐tLNPs encapsulating siRNA‐CKAP5 or siRNA‐NC were injected 7‐, 11‐, 14‐, 17‐ and 21 d post tumor inoculation (**Figure** **6**A). As a control, mock‐treated mice were injected with phosphate‐buffer saline (PBS) in a similar treatment regimen. On day 22 the mice were sacrificed and evaluated at various endpoints. Primarily, we witnessed that mice treated with *α*CD38‐tLNPs‐siRNA‐CKAP5 exhibited a significant decrease in the presence of MM cells in the spleen and BM both by IVIS live imaging (Figure 6B) and flow cytometry (Figure 6C,D). In the BM, while mock‐ and *α*CD38‐tLNPs‐siRNA‐NC‐treated mice had 18.09% and 16.96% occupancy of MM cells, in *α*CD38‐tLNPs‐siRNA‐CKAP5‐treated mice the occupancy reduced to 6.99%. The efficacy of *α*CD38‐tLNPs‐siRNA‐CKAP5 in the spleen is also reflected in the observed weight of the spleen across the treatment groups (Figure 6E,F). Altogether, this data indicates the ability of *α*CD38‐tLNPs to deliver therapeutic siRNA to MM cells residing both inside and outside the BM. In addition, as the levels of monoclonal protein are a hallmark clinical manifestation of disease burden in human MM disease, we examined the levels of the secreted light kappa chain in the serum. We noticed that treatment with *α*CD38‐tLNPs‐siRNA‐CKAP5 caused a substantial reduction to 825.5 ng mL^‐1^, compared with 1660 and 1972 ng mL^‐1^ in the *α*CD38‐tLNPs‐siRNA‐NC and mock‐treated group, respectively (Figure 6G). Collectively, our results demonstrate the broad potential of our *α*CD38‐tLNPs for treatment of MM for disseminated and BM‐associated cells in vivo and the ability of *α*CD38‐tLNPs‐siRNA‐CKAP5 to decrease disease burden in MM‐bearing mice.

## 3 Discussion

The past 5 years can be easily accepted as the most meaningful and revolutionary years in the field of RNA therapy, beginning with the approval of Onpattro siRNA‐LNP treatment for transthyretin amyloidosis^[^ ^10^ ^]^ and the success of the SARS‐CoV‐2 mRNA‐LNP as prophylactic vaccines, administered to billions around the world.^[^ ^11^, ^12^ ^]^ While RNA‐LNPs for hepatic indications and vaccines have successfully translated into the clinic, harnessing RNA therapy for hematological malignancies, and especially for B‐cell malignancies such as MM, is more complicated due to the limited delivery and the ineffective transfection of lymphocytes in vivo.^[^ ^7^, ^8^ ^]^

In this study, we report on a targeted delivery strategy utilizing LNPs to target both BM‐resident and disseminated MM cells. After screening different lipids and choosing L10 as an ideal lipid for transfecting MM cells in vitro, we generated targeted LNPs by conjugating an anti‐CD38 antibody to the surface of the LNPs. We validated the ability of the *α*CD38‐tLNPs to successfully disseminate to the BM niche and internalize into MM cells in vivo, and later their therapeutic potential was demonstrated upon injection into MM‐bearing mice. Treatment with *α*CD38‐tLNPs‐siRNA‐CKAP5 caused a significant reduction in the occupancy of MM cells in the BM and spleen, and decreased the overall disease burden, as indicated by the lower levels of monoclonal protein in the serum. Collectively, we believe our data shows the promising capabilities of targeted LNPs to deliver therapeutic RNA molecules into lymphocytes in non‐hepatic tissues.

The integration of a reliable disease model that resembles the human disease by mimicking the engraftment of MM cells to the BM is crucial for our study and the prediction it can give regarding the therapeutic capabilities of the *α*CD38‐tLNPs. Primarily, considering most existing examinations of RNA delivery to the BM are performed in healthy mice and with non‐targeted delivery systems,^[^ ^17^, ^28^, ^29^ ^]^ the translation of such systems towards any clinical application will necessitate defining a clearer therapeutic indication and require further evaluation in disease‐bearing mice. Moreover, the off‐target risks in applying non‐targeted delivery systems for lymphocyte‐related diseases are greater.^[^ ^16^ ^]^ An addition of a cellular targeting moiety can significantly improve the tLNPs’ targeting efficacy, increase retention at the target site, and, as we showed in our study, can decrease accumulation in non‐specific cells. Secondly, examination of RNA therapy for treatment of MM in existing murine xenograft models that lack homing of MM cells to their natural habitat in the BM is also problematic due to lack of indication regarding the ability of the evaluated drug to reach the tumor site. Although our newly established MM model is not located solely within the BM, it does allow the screening of different lipid formulations and targeting moieties to compare BM and cell‐specific retention. Also, to our knowledge, this is the first time that caudal artery injection was used to improve the homing of cells to the BM to generate a multiple myeloma mouse model. For example, our study showed that utilizing anti‐CD38 antibody as a targeting moiety significantly improved the specific arrival to MM cells compared with isotype control tLNPs. In addition, the employment of L10 as an ionizable lipid dramatically improved the arrival to the BM in comparison to MC3 ionizable lipid. Therefore, our model can be a useful tool for screening novel drugs for MM as it recapitulates the in vivo growth of MM cells in the BM. Furthermore, while xenograft MM mouse models usually lack clinical resemblance to the human disease,^[^ ^24^, ^25^ ^]^ this model shares many characteristic clinical symptoms of MM such as lytic bone lesions and secretion of monoclonal protein to the serum, symptoms which can be used to clinically evaluate the drugs. Yet, as with most complicated diseases, one model cannot fully resemble the human disease and an extensive preclinical evaluation of drugs for treatment of MM may require using other models as well.

Lastly, although our *α*CD38‐tLNPs‐siRNA‐CKAP5 were able to induce a significant therapeutic effect, combination therapy may be applied to improve it even further. MM patients are rarely treated with one therapeutic arm, and almost all treatment regiments are given as a combination of several drug classes due to the genetic and environmental variability involved in the disease.^[^ ^1^, ^30^, ^31^ ^]^ Combination therapy may be used by combining RNA therapy with other clinically approved drugs for MM, or by combining several different therapeutic RNA molecules in the same tLNPs to simultaneously affect different cellular pathways using the same LNP. Moreover, although we chose to knock down the expression levels of CKAP5 protein in this study, advancing this new therapeutic option to the clinic may require choosing a more tumor‐specific target such as *Bcl‐2* ^[^ ^32^ ^]^ or *Irf‐4*.^[^ ^33^ ^]^ Similarly, other targeting moieties like BCMA,^[^ ^34^, ^35^ ^]^ SLAMF7,^[^ ^36^, ^37^ ^]^ and CD138^[^ ^38^ ^]^ can be evaluated to allow the specific delivery to MM cells. Nevertheless, this therapeutic strategy opens new avenues for using RNA therapy as a novel drug class that has never been used before for treating MM and brings targeted LNPs and RNA‐based technologies closer to clinical application for all hematological malignancies.

## 4 Conclusion

MM is a common B‐cell malignancy in which MM cells inhabit the BM niche, making these malignant cells an elusive target for RNA therapy due to their location and challenging transfection. Here, targeted LNPs consisting of a novel ionizable cationic lipid were generated to target CD38, an overexpressed glycoprotein on MM cells, and encapsulated with siRNA to demonstrate their therapeutic potential. We confirmed the beneficial transfection efficiency of our LNPs over other ionizable lipids in vitro and validated their advanced targeting and delivery capabilities in a newly established xenograft MM murine model. Treating mice with only *α*CD38‐tLNPs‐siRNA to silence the expression of CKAP5, successfully reduced the occupancy of MM cells in the BM and lead to an overall decrease in disease burden, therefore indicating their clear antitumoral effects for MM. In addition, we demonstrated our novel MM mouse model can serve as a potent tool for evaluating therapeutic effects and BM retention abilities of newly developed drugs for treatment of MM, due to the high engraftment MM cells display in the BM. Overall, our study was the first to show the arrival of RNA therapy to MM cells residing in the BM of MM‐bearing mice and achieve a robust therapeutic effect. Therefore, we believe our results can prove the feasibility of implementing targeted LNPs for treatment of hematological malignancies. The potential future clinical application of RNA‐based technologies greatly relies on advancing the development of targeted delivery methods to GMP production and employing them to other non‐hepatic indications to ultimately revolutionize the field of cancer treatment.

## 5 Experimental Section

### Cell Culture

The human MM CAG cell line constitutively expressing luciferase (CAG‐Luc) was kindly provided by Dr. Anat Globerson‐Levine (Zelig Eshhar lab, Tel Aviv Sourasky Medical Center Ichilov).^[^ ^35^ ^]^ The human tumor cell lines Ovcar8 and HCT116 were purchased from American Type Culture Collection (ATCC). CAG‐Luc, Ovcar8, and HCT116 cells were sub‐cultured in fresh medium twice a week composed of Roswell Park Memorial Institute (RPMI) 1640 medium (Gibco) supplemented with 10% heat‐inactivated fetal bovine serum (FBS, Biological Industries), 1% 200 × 10^‐3^ m L‐glutamine (Gibco), and 1% 10,000 µg mL^−1^ penicillin‐streptomycin (Gibco). All cells were kept at 37 °C and 5% CO_2_ in a HERAcell 150i incubator (Thermo Scientific, USA) and routinely checked every two months for Mycoplasma contamination using EZ‐PCR Mycoplasma Test Kit (Biological Industries) according to the manufacturer's protocol.

### Primary Samples of MM Cells

Bone marrow samples were obtained from MM patients treated at the Rabin Medical Center (Beilinson Hospital, Petah Tikva, Israel) in accordance with Institutional Review Board‐Approved Informed Consent (0721‐17‐RMC). All experiments were carried out with the full, informed consent of the subjects. Malignant primary MM cells were isolated with whole blood and bone marrow CD138 MicroBeads kit (Miltenyi Biotec, USA) according to the manufacturer's protocol. Primary MM cells were grown in 24‐well culture plates (1 × 10^5^ cells per well) supplied with 1 mL of fresh medium composed of Iscove's Modified Dulbecco's Medium (IMDM, Gibco) supplemented with 10% FBS, 1% 200 × 10^‐3^ m L‐glutamine, and 10 000 µg mL^−1^ 1% penicillin/streptomycin, 1% non‐essential amino acids 100x solution (Gibco), 1% 100 × 10^‐3^ m sodium pyruvate (Biological Industries) and 0.1% 50 × 10^‐3^ m *β*‐mercaptoethanol (Gibco).

siRNAs: Chemically modified siRNA against human CKAP5 and MCL1, negative control (NC), Cy5‐labled siRNA‐NC were purchased from Integrated DNA Technologies (USA).
*siRNA‐CKAP5*:
sense strand: mUmArGmCrAmGrArGrUrUrAmUrGmArAmUrArArGmAA.
anti‐sense strand: rUrUmCrUrUrArUrAmUrUmCrAmUrArUrArArCrUrCrUrGmCrUmAmGmU
*siRNA‐MCL1*:
sense strand: mCmCrCmGrCmCrGrArArUrUmCrAmUmArAmUrUrUrArCrUmGT
anti‐sense strand: rArCmArGrUrArArAmUrUmArAmUrGrArArUrUrCrGrGrCmGrGmGmUA
*siRNA‐NC*:
sense strand: mCmUmUAmCrGmCmUrGrArGmUrAmCmUmCrGAdTsdT.
anti‐sense strand: rUrCrGrArArGmUrArCrUmCrArGrCrGmUrArArGdTsdT.

### Preparation of LNPs and Targeted LNPs

DLin‐MC3‐DMA (MC3), SM102, Lipid 10, and Lipid 14 were synthesized according to previously described method.^[^ ^17^, ^39^ ^]^ Cholesterol, DSPC (1,2‐ distearoyl‐sn‐glycero‐3‐phosphocholine), polyethylene glycol (PEG)‐DMG (1,2‐dimyristoyl‐rac‐glycerol), and DSPE‐PEG‐mal (1,2‐distearoyl‐sn‐glycero‐3‐phosphoethanolamine‐N‐[maleimide(polyethylene glycol)‐200](ammonium salt) were purchased from Avanti Polar Lipids Inc. Briefly, one volume of lipid mixture (ionizable cationic lipid, Cholesterol, DSPC and PEG‐DMG at 50:38.5:10:1.5 molar ratio) in absolute ethanol and three volumes of siRNA (1:6 mole N to P ratio RNA to ionizable lipid) in a 25 × 10^‐3^ m acetate buffer were injected into a NanoAssemblr microfluidic mixing device (Precision Nanosystems Inc.) at a combined flow rate of 12 mL min^−1^. For the preparation of targeted LNPs (tLNPs), lipid 10 or DLin‐MC3‐DMA, Cholesterol, DSPC, PEG‐DMG and DSPE‐PEG‐mal were mixed in the following ratio: 50:38:10:1.9:0.1. For preparation of LNPs‐Cy5‐LNPs, Cy5‐siRNA was used at 50% of total RNA amount. After LNPs generation, the particles were dialyzed against PBS (pH 7.4) for 24 h with two buffer exchanges to remove ethanol.

### Generation of Chemically Conjugated tLNPs

Isotype mouse IgG1 antibody (clone MOPC‐21, BioXCell) and anti‐human CD38 IgG antibody (clone THB‐7, BioXCell) were reduced with 1 × 10^‐3^ m dithiothreitol (Sigma‐Aldrich) and 5 × 10^‐3^ m EDTA (Sigma‐Aldrich) for 1 h at room temperature. Dithiothreitol was later removed by using 7K Zeba spin desalting column (ThermoFischer Scientific) according to manufacturer protocol and the reduced antibody was immediately added to the LNPs at a ratio of 1:40.7 antibody to LNPs (mg/mg) and incubated for 2 h at room temperature with gentle shaking and overnight at 4 °C. To remove free unconjugated mAbs, LNPs were loaded on CL4B Sepharose beads (Sigma‐Aldrich) and purified by gravity fed gel filtration chromatography column (BioRad Laboratories) using PBS as a mobile phase. The fractions were collected with a FC‐203B fraction collector (Gilson) and tLNPs fractions were collected and concentrated with 100K Amicon tubes (Millipore) to original volume.

### LNPs and tLNPs Characterization

Size distribution and PDI, and *ζ* potential were measured by Malvern Nano ZS *ζ* sizer (Malvern Instruments Ltd) in PBS or double‐distilled water, respectively. The encapsulation efficiency of siRNA was determined by Quant‐iT RiboGreen RNA assay (ThermoFischer Scientific) as previously described.^[^ ^39^ ^]^ Briefly. LNPs were either lysed or not with Triton X‐100, and after subtracting the blank measurement, the encapsulation efficiency (in percentages) was calculated as (1 − (non lysed LNPs/lysed LNPs)) × 100. For transmission electron microscopy analysis, 30 µL of aqueous solution containing naked or targeted LNPs was placed on a carbon‐coated copper grid, dried and analyzed using a JEOL 1200 EX transmission electron microscope.

### LNP Transfection

Cells were counted using trypan blue (Biological Industries). For transfecting CAG‐Luc MM cell line, LNPs were placed in 24‐well tissue plates in concentrations of 0.005–0.1 µg mL^−1^, and 2 × 10^5^ of cultured CAG‐Luc human MM cells with 1 mL growing medium were later added to the wells. For transfecting Ovcar8 and HCT116 cell line, 1 × 10^5^ cells were placed in 12‐well tissue plates or 0.5 × 10^5^ cells were placed in 24‐well tissue plates, respectively, with 1 ml of growing medium overnight. LNPs were then added in concentrations of 0.005‐0.1 µg mL^−1^. All cells were incubated with the treatments in standard culture conditions for 24 to 72 h. For transfecting primary MM cells ex vivo, tLNPs were placed in 24‐well tissue plates in concentrations of 1–5 µg mL^−1^, and 2 × 10^5^ of primary MM cells 1 mL were added to the wells. The cells were then moved to 4 °C for 30 min, centrifuged at 1300 rpm for 5 min, and the medium was replaced with fresh media to remove unbound tLNPs. Cells were then moved to the incubator at 37 °C and grown in for 48 h.

### Cell Viability Studies

For determining cell viability of CAG‐Luc, Ovcar8, and HCT116 cells were collected 72 h after LNP transfection using XTT Cell Proliferation kit (Biological Industries), according to manufacturer's instructions. Plates were incubated for 3 h at 37 °C in dark conditions are read in colorimetric plate reader (BioTek). For determining cell viability of primary MM cells were collected 48 h after LNP using CellTiterGlo cell viability assay (Promega) according to manufacturer's protocol and read in luminometer (GloMax Navigator, Promega).

### LNPs Binding and Internalization Assessment

For binding assessment, 1 × 10^6^ CAG‐Luc cells were washed twice with cold PBS and then incubated with 100 µL of full medium containing 1 µg of *α*CD38‐tLNPs or iso‐tLNPs for 30 min at 4 °C. Then, cells were washed twice with PBS and incubated with 100 µL donkey anti‐mouse IgG PE (1:100, Jackson Immuno Research) diluted in PBS containing 1% BSA for 30 min at 4 °C. Cells were then washed and analyzed by flow cytometry. For competitive binding assessment, the same procedure was performed with and without pre‐coating of the cells with 1 µg of free anti‐CD38 antibody diluted in PBS containing 1% BSA for 30 min before adding the Cy5‐siRNA‐tLNPs. For internalization assessment, 1 × 10^6^ cells were washed twice with cold PBS and then incubated with 100 µL of full medium containing 1 µg of *α*CD38‐tLNPs or iso‐tLNPs encapsulating Cy5‐siRNA for 30 minutes at 4 °C. Then, the cells were washed twice with PBS, suspended in 200 µL of full media and inserted for 6 or 0 h (as control) into the incubator at 37 °C. Afterwards, the cells were washed, stained with pre‐warmed Hoechst in full medium (1:5000, Sigma‐Aldrich) for 30 min, washed twice with PBS, and stained with anti‐human CD44 Alexa488 (Biolegend) for 30 min in room temperature. They were then washed, resuspended in 100 µL of PBS and subjected to confocal microscopy analysis. All pictures were obtained on live cells using Nikon Eclipse C2 configured with NI‐E microscope and processed with NIS‐elements software using x40 objective magnification (Nikon).

### Real‐Time PCR Analysis

3 × 10^5^ cells were collected 24 h after LNP transfection. Total RNA was extracted with EZ‐RNA II total RNA isolation kit (Biological Industries) according to manufacturer's protocol and quantified by NanoDrop (ThermoFischer Scientific). Later, cDNA was synthesized with qScript cDNA synthesis kit (Quanta Bio, M, USA) according to manufacturer's protocol. cDNA was diluted 1:3 in nuclease free water (IDT) and real‐time PCR was carried with Fast SYBR® Green Master Mix (Applied Biosystems, ThermoFischer Scientific) and ABI StepPlusOne instrument (Applied Biosystems, USA).

*Human CKAP5 Primers*: Forward: 5’ TGGTGGCTTTGGCAGCAAA.
Reverse: 5’ TCCAAGATGGTTGGCACAACAT
*Human eIF3C Primers*: Forward: 5’ ACCAAGAGAGTTGTCCGCAGTG
Reverse: 5’ TCATGGCATTACGGATGGTCC

### Cell Cycle Arrest Assay

5 × 10^5^ cells were collected 36 h post‐transfection and washed twice with ice‐cold PBS. The cells were then fixed with 75% cold ethanol for 30 min, washed twice with cold PBS, and incubated for 12 min at 37 °C in 220 µL of PBS with DAPI (2‐(4‐amidinophenyl)‐6‐indolecarbamidine dihydrochloride 15 mg mL^−1^; Merck) and 0.01% Triton‐X100 (Sigma‐Aldrich). Fluorescence was measured by flow cytometry (CytoFLEX and the CytExpert software, Beckman Coulter, USA). Analysis was done with FlowJo software (FlowJo LLc, USA).

### Confocal Imaging of Cell Cycle Arrest

1.5 × 10^6^ cells were collected 36 h post‐transfection with 0.1 µg LNPs and washed twice with PBS. The cells were then moved to a 24‐well plate containing glass cover slips pre‐coated with poly‐L‐lysine (Sigma‐Aldrich), centrifuged for 8 min at 800 g, and then PBS was replaced immediately with 150 µL of fixation buffer composed of: 4% paraformaldehyde (Sigma‐Aldrich), 8% glutaraldehyde (Sigma‐Aldrich) and 10% Tritox‐X100 (Sigma‐Aldrich). After 20 min of incubation at room temperature, the cells were washed 3 times with sodium borohydride (Sigma‐Aldrich), incubated at room temperature for 15 min and blocked for 1 h with 3% BSA (MP Biomedicals). The cells were then washed and added with 50 µL of PBS with 0.1% Triton X‐100 containing mouse anti‐human *α*Tubulin antibody (1:100, clone: DM1A, Sigma‐Aldrich) for 1 h incubation in a humid chamber. Afterward, cells were washed 3 times with PBS and incubated with 50 µL of PBS with 0.1% Triton X‐100 containing donkey anti‐mouse IgG Alexa488 (1:400, Jackson Immno Research) for 1 h at a humid chamber. After one wash with PBS, the cells were stained with 200 µL of Hoechst diluted in full medium, incubated for 2 minutes in dark conditions, and washed twice with PBS. Lastly, the coverslips were loaded on glass slides using 15 µL of lab‐made mounting fluid and left overnight at dark conditions. The samples were then read and analyzed using Leica SP8 confocal microscope (Leica Microsystems, Germany).

### Animal Experiments

All animal protocols were approved by Tel Aviv University Institutional Animal Care and Usage Committee (TAU‐LS‐IL‐2302‐119‐5) and in accordance with current regulations and standards of the Israel Ministry of Health. The mice were housed and maintained in laminar flow cabinets under specific pathogen‐free conditions.

### Xenograft MM Mouse Model Establishment

Six‐ to eight week old female R2G2 (B;129‐Rag2^tm1Fwa^Il2rg^tm1Rsky^/DwIHsd, Envigo, Rehovot, Israel) mice were injected with 1 × 10^6^ CAG‐Luc via the caudal artery (C.A).^[^ ^26^ ^]^ The mice were monitored biweekly for weight loss and tumor progression. For tumor progression monitor, the mice were injected intraperitoneally with 15 mg of XenoLight D‐Luciferin (122 799, PerkinElmer Inc.) and imaged by IVIS bioluminescence imaging system (IVIS SpectrumCT. PerkinElmer Inc.). Twenty‐four days after C.A injection, the mice were sacrificed, their liver, spleen, lungs, and BM were imaged by IVIS. Their BMs were harvested and processed into single‐cell suspensions for further analysis of the engraftment of human MM cells in the BM. Briefly, femurs were flushed with 1.5 mL of PBS, strained through a 100 × 10^‐6^ m, stained with 1 µg anti‐human CD29 PE antibody (clone TS2/16, Biolegend) and a negative signal of anti‐mouse CD45 APC (clone 30‐F11, Biolegend) for 30 min in 4 °C, and analyzed by flow cytometry. Determination of appointed time for mice sacrifice was based on evaluation of whole‐body luciferase signal obtained from IVIS combined with a physical evaluation of the mice, since they showed no behavioral changes, didn't have hind‐leg paralysis or lost body weight as the disease progressed.

### Histology Staining

Mice were sacrificed after 24 d, femurs were extracted, fixed with Fixation Buffer (554655, BD Biosciences), processed and analyzed for H&E stain or IHC with anti‐TRAP*α* (Novus Biologicals, Cat NBP1‐86912, USA) by Gavish Research Services (Nes Ziona, Israel).

### Whole Body CT and Micro‐CT

Twenty‐four days post tumor inoculation, mice were imaged with small animal CT (PET/SPECT/CT system, MILabbs, The Netherlands). Later, the mice were sacrificed, femurs were extracted, fixed with Fixation Buffer for 48 h, and transferred to 70% ethanol. The femurs were then imaged with micro‐CT (XT H 225 ST X‐Ray Micro‐Computed Tomography (Micro‐CT) system, Nikon).

### Quantification of Human Light Kappa Chain

The levels of human light kappa chain in murine serum were measured to assess whole body disease burden. Serums were collected during animal studies and stored at ‐80 °C and analyzed by ELISA in duplicates. Plates were coated with 50 µL per well with mouse anti human kappa light chain (2 µg mL^−1^, clone TB28‐2, Biolegend) and incubated overnight at 4 °C. The plates were washed once with PBS containing 0.5% of Tween 20 and 150 µL of PBS containing 1% of BSA was added to the wells for a 2 h incubation at 37 °C. After one wash with PBS/Tween 20, 50 µL of diluted sample or Rituximab IgG (kindly provided by Prof. Itai Benhar, Tel Aviv University), were added to the plates and incubated for 2 h at room temperature. After washing 3 times with PBS/Tween 20, 50 µL of goat anti‐human kappa light chain HRP conjugated antibody (1:20,00, Bethyl, Fortis Life Science) was added to the wells for a 2 h incubation at room temperate. The plates were then washed 3 times with PBS/Tween 20, 50 µL of TMB solution (Millipore) was added as substrate, and the reaction was stopped by adding 2 m H_2_SO_4_. Results were analyzed by reading absorbance 450 nm read in colorimetric plate reader (Synergy HT, Biotek). The standard curve was linear between 2.34 and 150 ng mL^−1^, and samples were diluted to a concentration within this range.

### Biodistribution Studies

Sixteen days post tumor inoculation, mice were imaged by IVIS bioluminescence imaging system and randomized to groups of 3–4 mice based on their disease progression. The mice were then injected retro‐orbitally with 1 mg kg^−1^ of *α*CD38‐tLNPs or iso‐tLNPs comprised of L10 or MC3 and encapsulating Cy5‐siRNA. 4 and 24 h later, the mice were sacrificed, and major organs (liver, spleen, kidneys, and femurs) were imaged by IVIS bioluminescence imaging system. Liver, spleen, and BMs were harvested and processed into single‐cell suspensions for further analysis by flow cytometry.

### In Vivo Safety Study

Female, eight week old C57Bl/6 mice (Envigo laboratories) were injected with iso‐tLNPs at a dose of 1 mg kg^−1^ and sacrificed 24 h later. Blood was collected and analyzed by AML Israel for biochemistry (Cobas‐6000).

### Efficacy Studies

Six‐ to eight‐week‐old female R2G2 mice were injected with 1 × 10^6^ CAG‐Luc via the caudal artery (C.A) and imaged by IVIS bioluminescence imaging system for randomization of 3 mice per group after 7 d. The mice were injected retro‐orbitally with 200 µL containing 1 mg kg^−1^ of *α*CD38‐tLNPs encapsulating either siRNA‐NC or siRNA‐CKAP5, or injected with PBS, at days 7, 11, 14, 17, and 21. Twenty‐two days after tumor inoculation the mice were sacrificed, serums were collected, and major organs (liver, spleen, and femurs) were imaged by IVIS bioluminescence imaging system. The BM and spleen were harvested and processed into single‐cell suspensions for further analysis by flow cytometry.

### Statistical Analysis

Statistical analysis for comparing two experimental groups was performed using two‐sided Student's *t* tests. In experiments with multiple groups, one‐ or two‐way analysis of variance (ANOVA) with a Tukey correction was used to calculate differences among multiple populations. Analyses were performed with Prism 7 (GraphPad Software). Differences are labeled as * for *P* ≤ 0.05, ** for *P* ≤ 0.01, *** for *P* ≤ 0.001, **** for *P* ≤ 0.0001.

## Conflict of Interest

D.P. declares the following competing financial interest(s): D.P. receives licensing fees (to patents on which he was an inventor) from, invested in, consults (or on scientific advisory boards or boards of directors) for, lectured (and received a fee) or conducts sponsored research at TAU for the following entities: ART Biosciences, BioNtech SE, Eleven Therapeutics, Kernal Biologics, Merck, Newphase Ltd., NeoVac Ltd., RiboX Therapeutics, Roche, SirTLabs Corporation, Teva Pharmaceuticals Inc. All other authors declare no competing financial interests.

## Supporting information

Supporting Information

## Data Availability

The data that support the findings of this study are available from the corresponding author upon reasonable request.
