# Supplementary material for: Delivery of Therapeutic RNA to the Bone Marrow in Multiple Myeloma Using CD38‐Targeted Lipid Nanoparticles
Source: Adv Sci (Weinh). 2023 May 12;10(21):2301377. doi: 10.1002/advs.202301377 (PMC10375190; doi:10.1002/advs.202301377)
Supplement: Supplementary file 1 — Supporting Information [file ADVS-10-2301377-s001.pdf]

ADVANCED SCIENCE
Open Access

Supporting Information

for *Adv. Sci.*, DOI 10.1002/advs.202301377

Delivery of Therapeutic RNA to the Bone Marrow in Multiple Myeloma Using CD38-Targeted Lipid Nanoparticles

*Dana Tarab-Ravski, Inbal Hazan-Halevy, Meir Goldsmith, Lior Stotsky-Oterin, Dor Breier, Gonna Somu Naidu, Anjaiah Aitha, Yael Diesendruck, Brandon D. Ng, Hagit Barsheshet, Tamar Berger, Iuliana Vaxman, Pia Raanani and Dan Peer**

Delivery of Therapeutic RNA to the Bone Marrow in Multiple Myeloma using CD38-Targeted Lipid Nanoparticles

*Dana Tarab-Ravski, Inbal Hazan-Halevy, Meir Goldsmith, Lior Stotsky-Oterin, Dor Breier, Gonna Somu Naidu, Anjaiah Aitha, Yael Diesendruck, Brandon D. Ng, Hagit Barsheshet, Tamar Berger, Iuliana Vaxman, Pia Raanani, Dan Peer**

Supporting information

Supporting information is available from the Wiley Online Library or from the author.

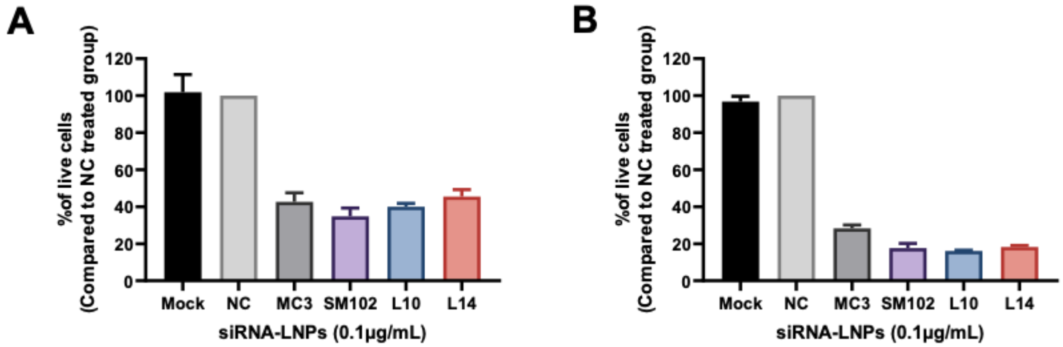

Figure 1S: Evaluation of transfection efficiency on Ovcar8 and HCT116 tumor cell lines. A) Ovcar8, and (B) HCT116 cell viability 72 hours post-transfection with either PBS, LNPs-siRNA-CKAP5, or LNPs-siRNA-NC in concentration of 0.1 µg mL^{-1} total RNA. Cell viability percentages were normalized to cells treated with LNPs-siRNA-NC in 0.1 µg mL^{-1} of total RNA. Data shown are means of three independent experiments ±SD.

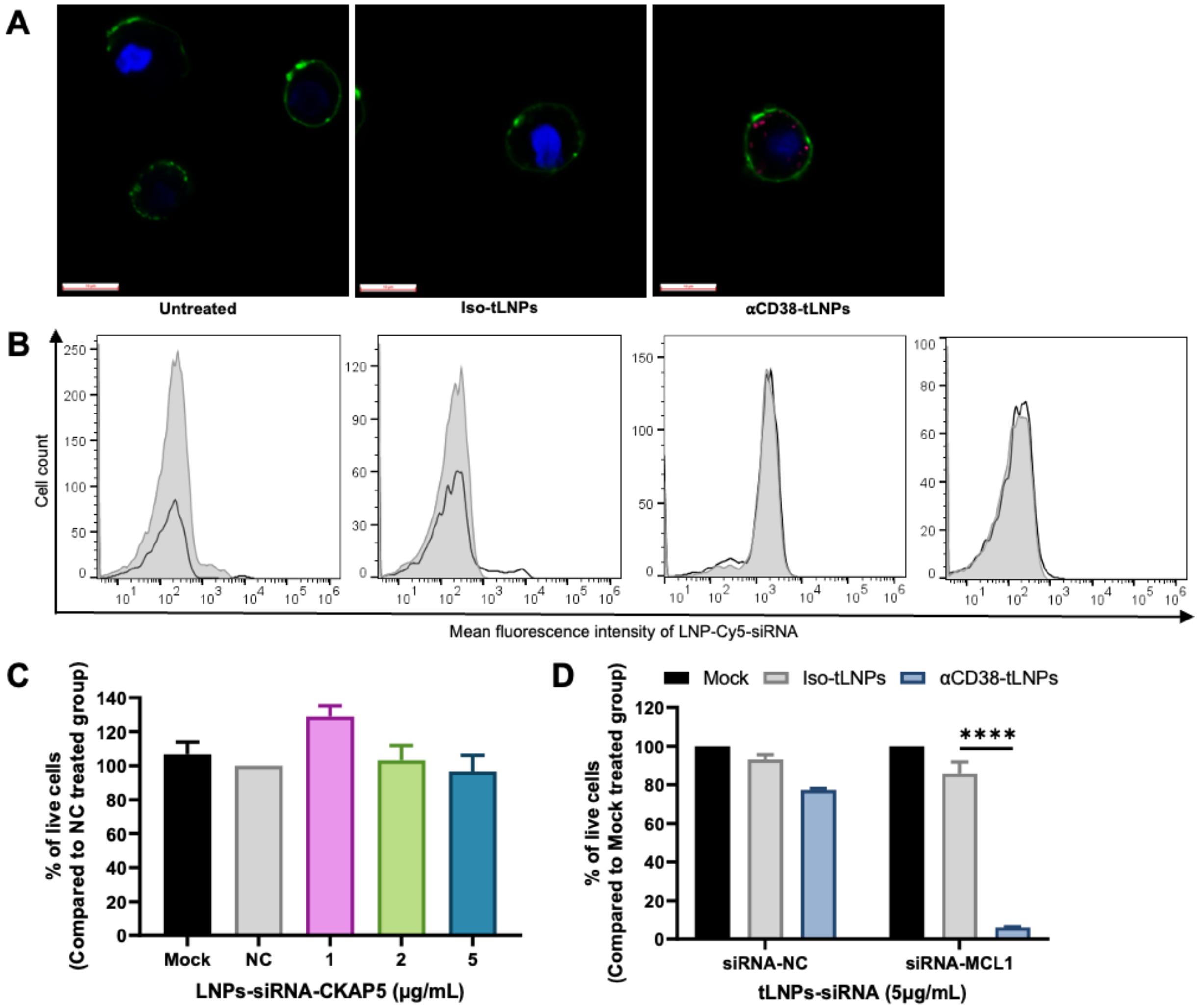

Figure 2S: Internalization and therapeutic effect of targeted LNPs on primary MM cells *ex vivo*. A) Representative live confocal images showing the internalization of αCD38-tLNPs-Cy5-siRNA (shown in red) into primary MM cells. DNA was stained with Hoechst 33342 (blue) and membranes were stained with an anti-CD44 antibody conjugated to Alexa488 (green). (Scale bar = 10μm). B) Representative diagrams showing lack of binding of naked LNPs-Cy5-siRNA (black line) into primary MM cells (n=4 patients). Untreated primary MM cells are shown in grey. C) Representative CellTiterGlo cell viability assay of primary MM cells treated with mock or naked LNPs encapsulating either siRNA-NC or siRNA-CKAP5 in a concentration of 1, 2, and 5 μg mL^{-1} total RNA. Bar chart representing % of cell viability normalized to siRNA-NC treated cells in a concentration of 5 μg mL^{-1} total RNA. D) Representative CellTiterGlo cell viability assay of primary MM cells treated with mock, iso-tLNPs, or αCD38-tLNPs encapsulating either siRNA-NC or siRNA-MCL1 in a concentration of 5 μg mL^{-1} total RNA. Bar chart representing % of cell viability normalized to mock-treated cells. Two-way analysis of variance (ANOVA) with Tukey multiple comparison test was used to assess the significance. ****$P<0.0001$.

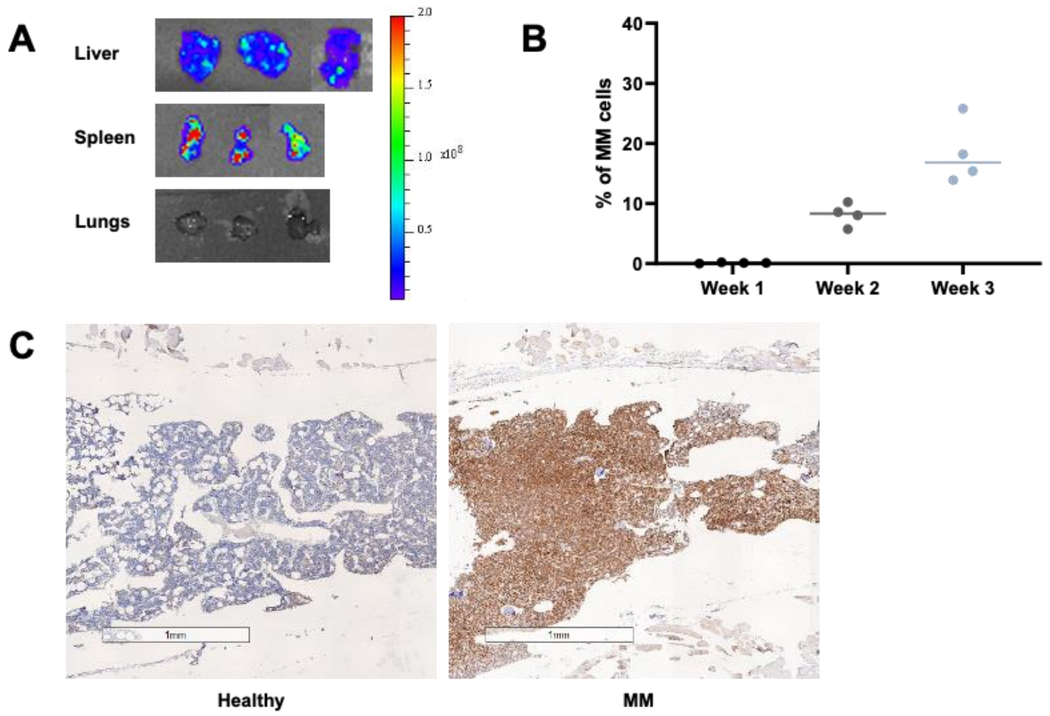

Figure 3S: Establishment of novel xenograft MM mouse model. A) Bioluminescence imaging of the liver, spleen, and kidneys 24 days after injection with CAG-Luc cells to mice, n=3 / group and experiment was repeated 3 times. B) Percentages of MM cells engraftment in the femurs at 1, 2, and 3 weeks after tumor post tumor inoculation, n=4 / group. C) Representative images of healthy (left) and MM-bearing mice of TRAP immunohistochemistry stain of femurs 24 days after injection with CAG-Luc cells to mice (Scale bar = 1mm), n=3 / group.

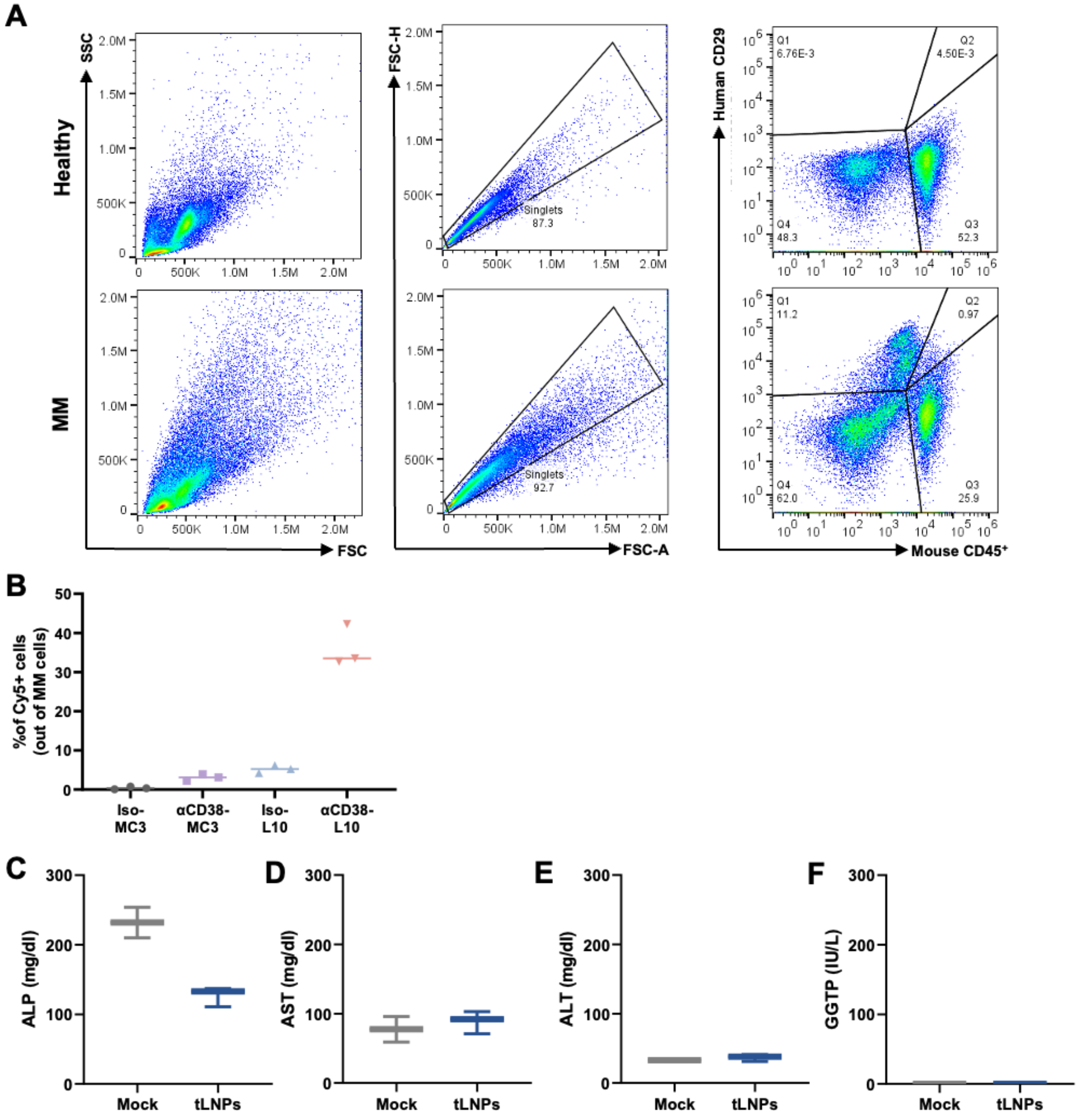

Figure 4S: In vivo biodistribution of targeted LNPs in MM-bearing mice. A) Gating strategy of human MM cells and mouse CD45 positive cells in representative healthy (upper row) and MM-bearing mice (lower row), n=3 / group. Left column represents the forward scatter (FSC) and side scatter (SSC), middle column represents single cells by forward scatter area (FSC-A) to forward scatter height (FSC-H), and right column represents single cells stained with anti-human CD29 PE and anti-mouse CD45 Alexa 488. B) Percentages of Cy5-positive MM cells in the BM, spleen, and liver 24 hours after injection of tLNPs as analyzed by flow cytometry, n=3 / group. C-F) Serum levels of liver enzymes. No significant elevation was detected in the tLNP-treated mice compared to non-treated mice (Unpaired T-test was used to assess the significance), n=2 per mock-treated group, n=3 per tLNPs-treated group.

Conjugation efficiency of targeted LNPs

Conjugation efficiency of antibody to LNPs for generation of targeted LNPs is 32.49±8.2%. This evaluation was conducted for 3 individual preparations of targeted LNPs. The conjugation efficiency of targeted LNPs was determined by quantifying amount unconjugated antibodies remaining in the flow through of the gel filtration chromatography column following conjugation. Briefly, after collection of the targeted LNPs, the flow through was collected, concentrated by 10K Amicon tubes (Millipore), and the amount of unconjugated antibody was quantified by BCA kit (Thermo Fisher Scientific). Conjugation efficiency (in percentage) was calculated as:

$$100 - \left[\left(\frac{amount\ of\ unconjugated\ antibody\ after\ conjugation}{amount\ of\ antibody\ before\ conjugation} \right) * 100 \right]$$
